# Improvement of Autism Symptoms After Comprehensive Intensive Early Interventions in Community Settings

**DOI:** 10.1177/1078390320915257

**Published:** 2020-04-23

**Authors:** Nils Haglund, SvenOlof Dahlgren, Maria Råstam, Peik Gustafsson, Karin Källén

**Affiliations:** 1 Nils Haglund, PhD, Lund University, Lund, Sweden; 2 SvenOlof Dahlgren, PhD, University of Gothenburg, Gothenburg, Sweden; 3 Maria Råstam, MD, Lund University, Lund, Sweden; University of Gothenburg, Gothenburg, Sweden; 4 Peik Gustafsson, MD, Lund University, Lund, Sweden; 5 Karin Källén, PhD, Lund University, Lund, Sweden

**Keywords:** autism symptoms, ADOS scores, intervention program

## Abstract

**Background:**

Preschool children with autism in southern Sweden participated in a comprehensive Naturalistic Developmental Behavioral Intervention (NDBI) program.

**Aims:**

To evaluate the ongoing NDBI program by comparing the pre- and postintervention outcomes in terms of improved autism symptom severity.

**Method:**

The improvement of Autism Diagnostic Observation Schedule (ADOS-R) test results between baseline and evaluation among children participating in the NDBI program (*n* = 67) was compared with the results among children receiving community treatment as usual (*n* = 27) using analysis of covariance.

**Results:**

The study showed that children in the NDBI group improved their ADOS-R total scores between baseline and evaluation (−0.8 scores per year; 95% CI [−1.2, −0.4]), whereas no improvement was detected in the comparison group (+0.1 scores per year; 95% CI [−0.7, +0.9]). The change in the NDBI group versus the change in the comparison group was statistically significant after adjusting for possible confounders as well. Children in the NDBI group also significantly improved their ADOS severity scores, but the scores were not significantly different from those of the comparison group.

**Conclusions:**

The results from the current naturalistic study must be interpreted cautiously, but they do support earlier studies reporting on improvement of autism symptoms after early intensive interventions. Results from observational studies are difficult to interpret, but it is nevertheless of uttermost importance to evaluate costly autism intervention programs. The results do indicate that children with autism benefit from participating in early comprehensive intensive programs.

## Introduction

The increase in early detection and diagnosis of children with autism spectrum disorders (ASDs) in the past two decades has challenged child and youth habilitation centers to offer and provide affected children and their parents the best and most appropriate treatment and support, respectively. During the past 15 to 20 years, the diagnostic criteria for autism, and diagnostic substitution, respectively, have widened the autism spectrum. These factors are believed to be the main reason for the worldwide significant increase in prevalence of autism (Fombonne et al., 2009; King & Bearman, 2009; Lundström et al., 2015; Rice et al., 2012). The expanded spectrum inevitably results in an increasing heterogeneity among children with ASD diagnoses, which cause problems in recommendation for intervention and treatment. Many aspects must be considered when designing an individual intervention program. There is not one pedagogy that fits all under the expanded autism spectrum umbrella (Roane et al., 2016). There is some, albeit limited, evidence that early onset of intervention, in general, indicates better outcomes in cognitive and adaptive functioning in children with autism (Howlin et al., 2009). An early intervention onset requires an early detection of ASD, and a significant trend toward such an early detection has been reported (Chawarska et al., 2007; Costanzo et al., 2015; Kleinman et al., 2008; Lord et al., 2006; Rice et al., 2012).

The development of evidence-based strategies to treat children with autism was first established in the 1970s and 1980s, when the publication of Lovaas (1987) -controlled study reported that early intervention based on applied behavior analysis (ABA) significantly increased cognitive abilities as reflected on IQ tests (Lovaas, 1987). A follow-up study further showed that initial gains through early intensive intervention could persist into adolescence (McEachin et al., 1993). The importance of parents being involved in the intervention program was observed earlier (Berkowitz & Graziano, 1972), and reports from different programs based on ABA techniques (Vismara & Rogers, 2010) have provided further evidence of this observation. Since Lovaas’s first discrete trial training (Lovaas, 1987), new approaches to strategies to increase the children’s motivation for participating in training programs have been developed (Dunlop & Koegel, 1980; Koegel et al., 1987; Schreibman et al., 1982). Strategies such as pivotal response training (Pierce & Schreibman, 1995; Stahmer, 1995) incorporate natural rather than artificial reward systems and provide the children more opportunities to choose materials and tasks during the training session. The importance of developing a milieu that offers training in interaction with parents, siblings, and preschool staff has also been stressed, and it is today regarded to be the most useful factor when establishing a comprehensive intervention program for children with ASD (Cohen et al., 2006; Eikeseth et al., 2002; Prizant et al., 2003; Toth et al., 2006). Several approaches have been introduced to integrate methods based on developmental sciences in ABA intervention programs, implemented by both clinicians and parents (Dawson & Bernier, 2013). In recent years, different comprehensive learning programs aimed to engage children with ASD have been gathered under the label of “Early Intensive Behavior Intervention” (EIBI) programs. EIBI programs are manual-based intensive programs, and they target a comprehensive range of skills for training, practice, and generalization. In a recently published meta-analysis, (Fuller & Kaiser, 2019; Nahmias et al., 2019), authors found evidence for positive outcome in social communication, after early interventions. Nahmias et al. (2019) found EIBI programs associated with universities superior to only community-based programs evaluating cognitive and adaptive outcome. In the latest update of previous Cochrane Review of EIBI for ASD (Reichow et al., 2018), five relevant studies were included. Only one study was randomly designed as a randomized controlled trial study. The other four were described as quasi-randomized controlled trials or controlled clinical trials to compare EIBI treatment. This review found some, but weak, evidence that children receiving EIBI treatment performed better than children in a comparison group on scales of adaptive behavior after 2 years of treatment. When evaluating the severity of autism symptoms, the review found the quality of evidence as very low for positive outcome.

The importance of merging applied behavioral and developmental sciences has been stressed, and “Natural-istic Developmental Behavioral Interventions (NDBI),” involving shared control between child and therapist in natural settings, have been found to be efficacious and are supported by a large body of evidence (Dawson et al., 2010; Ryberg, 2015; Schreibman et al., 2015). The effects of miscellaneous recent intervention programs have been evaluated. Shire et al. (2017) found significantly improved social skills among children randomized to an intensive intervention program involving parents and preschool staff (the JASPER [Joint Attention, Symbolic Play, Engagement, and Regulation] intervention) compared with community ABA treatment as usual. However, neither Fernell et al. (2011) nor Spjut Jansson et al. (2016) detected any association between improvement of adaptive skills and level of intervention intensity among children with ASD participating in different comprehensive NDBI programs in Sweden.

The growing number of children with ASD diagnosed at an early age challenge the communities to offer evidence-based intervention programs to new groups of children (and their families). The setting of a modern integrated NDBI treatment program involves not only staff at the child habilitation center but also staff at health services and preschools. Such an integrated NDBI program is established at all child and youth habilitation centers in Skåne County in southern Sweden. These centers are responsible for intervention, support, and habilitation for all children with ASD and their families. In light of the increasing number of children diagnosed with ASD, the resource consuming NDBI is increasingly questioned by health authorities, and a thorough evaluation of the interventions is urgent.

The aim of the present study was to evaluate an ongoing, comprehensive NDBI program for children with ASD, implemented in the child’s milieu at home and in the preschool. The hypothesis was that children, 3 to 7 years of age, who participated in the NDBI program offered to all children with autism in the south of Sweden, had lower Autism Diagnostic Observation Schedule (ADOS) score after accomplished program than they had at base line and that the ADOS scores improved more among children who participated in the NDBI program than the corresponding ADOS scores did among children with autism who received conventional habilitation services.

## Method

### Participants

**Figure 1.**
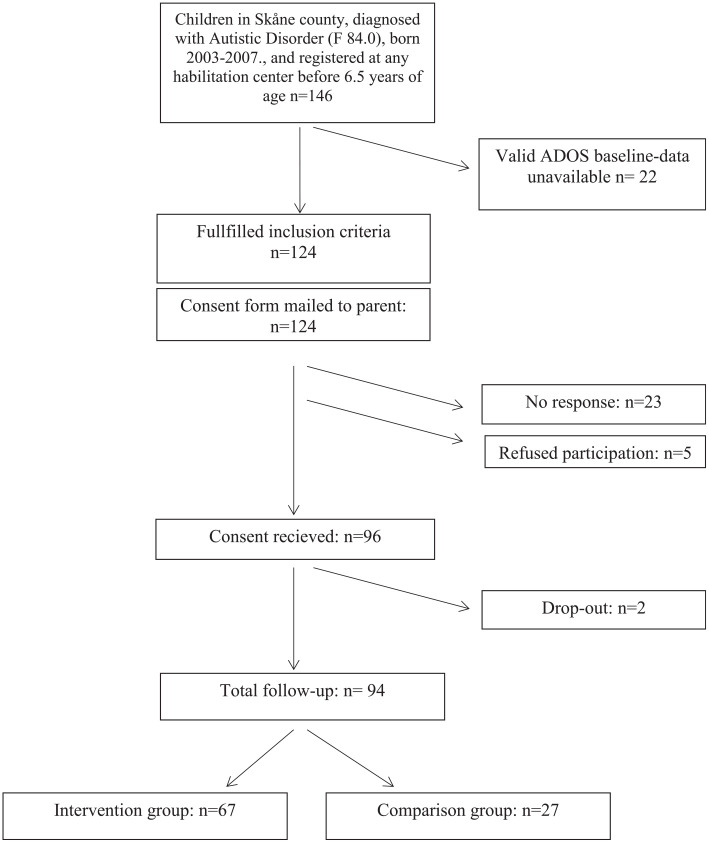
Flowchart showing the formation of the study groups.

The current evaluation included children born between 2003 and 2007, who were diagnosed with autistic disorder (*Diagnostic and Statistical Manual of Mental Disorders*–Fourth Edition [*DSM-IV*]; American Psychiatric Association, 1994); 299.00/International Classification of Diseases–10th Revision (ICD-10); F 84.0, and referred to any of the child and youth habilitation centers in Skåne before 6.5 years of age for intervention and support. After exclusion of children without valid baseline ADOS data and/or written consent, and two dropouts, 94 children remained for analyses (Figure 1).

All parents were offered a 2-day-long education session, summarizing today’s knowledge of autism and introducing the concepts of the NDBI program. Sixty seven children whose parents accepted the offered program constituted the intervention group, whereas children whose parents (for any reason) were not interested in the offered intervention program constituted the comparison group (*n* = 27; Figure 1). All 67 children in the intervention group were enrolled in the NDBI program at their local habilitation center within 3 months after diagnosis.

#### Intervention: The NDBI Program

The intervention program was established in the Skåne county in 2004, based on recommendations from the Swedish report “Comprehensive Intensive Early Interventions” (Bromark et al., 2004), similar to the NDBI described by Schreibman et al. (2015). Schreibman et al. describes 13 evidence-based features common for and central to different NDBI interventions. Among these, “Manualized Practice,” “Fidelity of Implementation Criteria,” “Individualized Treatment Goals,” “Ongoing Measurement of Progress,” “Child-Initiated Teaching Episodes,” “Environmental Arrangement,” “Natural Reinforcement and Related Methods for Enhancing Motivation of the Child,” “Use of Prompting and Prompt Fading,” “Balanced Turns Within Object or Social Play Routines,” “Modeling,” “Adult Imitation of the Child’s Language, Play, or Body Movements,” and “Broadening the Attentional Focus of the Child” all were a part of the NDBI program package offered in the Skåne region. The only feature described by Schreibman that was not always utilized was “Three Part Contingency,” which requires that children can adapt the ABA treatment (antecedent-response-consequence) technique. The NDBI program was individualized to each child by experienced ABA specialists with different backgrounds as psychologists, speech pathologists, or special education teachers, in cooperation with parents and preschool staff. All participating parents and preschools trainers were educated in autism and ABA techniques before intervention. The preschool trainers were given special education in the applied ABA method, including theoretical lessons in autism and ABA, and workshops to gain skills and knowledge on how to perform a wide range of exercises included in the NDBA program. Most preschool trainers had long experience working with children with autism. Before starting the intervention program, each child was assessed to find the child’s strengths and disadvantages. According to this assessment, a treatment plan for 2 years was agreed upon, and the plan was revised every 6 months. Every child with autism in the intervention group was assigned two ABA specialists to ensure continuity and quality over a 2-year period. The ABA specialists designated to each child developed the individualized training program and instructed the parents and preschool trainer. The parents and preschool trainer were expected to follow the program with the individualized exercises for a 2-week period after which the intervention team would meet and evaluate the results. New tasks and exercises were introduced if the old ones were satisfactorily implemented. The exercises varied from simple tasks where the child was asked to point to his or her nose, eyes, mouth, and/or hair; to name familiar objects; to enumerate objects in a picture; and to tell his or her last name, to more complicated tasks, including participating in social interpersonal communication.

The intensity was calculated for 15 to 25 hours each week at home and at the preschool all together. During the second year, the support could be less intensive (scheduled appointments with the ABA staff every 3 or 4 weeks), but still, parents and preschool trainer were expected to work 15 to 25 hours with the child each week. Every 6 months, the NDBI program was evaluated and the intervention intensity was assessed.

#### Interventions in the Comparison Group

Parents of children in the comparison group were given support according to an agreement between parents and staff at their local habilitation center. Thus, the interventions received by the children in this group were not based on any intervention program developed at the habilitation centers but were adapted to each child’s (and family’s) needs. The support consisted of different kinds of targeted types of training (speech and language training, toilet training, home support, or other forms of time-limited efforts) that the parents asked for. Some of the interventions that children in the intervention group received were also available for children in the comparison group. For children in the comparison group, there were no agreements with the parents regarding scheduled time for weekly activities, and no involvement of preschool staff was postulated. Also, parents in the comparison group were not educated in the NDBI program and were not contracted to work with the individualized exercises at home. The number of appointments with personnel at the local habilitation center varied with the individual requirements, in average two to three appointments during each 6-month period.

#### Preintervention Assessment (Baseline Data)

Data on the autism severity of all children, according to ADOS-R scores (Lord et al., 2000) and cognitive development, were collected from the prior diagnostic evaluation performed at any of the child psychiatric clinics in Skåne county. The ADOS is a standardized observation scale first developed in the United States, Canada, and the United Kingdom, but over the years internationally used and translated to more than 20 languages. It is designed to assess important social–communicative behavior as well as stereotypic and repetitive features, according to ICD-10 and *DSM-IV*/*DSM-5* criteria for autism/ASD. All items assessed in the semistructured interaction between the observed person and the examiner are coded from 0 (no abnormality related to autism) to 2 (definitive abnormality). The ADOS is developed to be appropriate for individuals with suspected ASD, from a mental and chronological age of 12 months to adulthood. Over the years, the ADOS has been developed from three to five modules, with algorithms, to minimize the influence of language and age for the manifestation of ASD symptomatology (Zander, 2015). The ADOS-R assessments in our study were performed by experienced ADOS-certified psychologists. When possible (*n* = 29), cognitive development was assessed by Wechsler Preschool and Primary Scale of Intelligence–Third Edition) (WPPSI-III; Wechsler, 2005). Otherwise (*n* = 38), a developmental age was calculated from information retrieved from Griffiths Mental Development Scales (Alin-Åkerman & Nordberg, 1991), Psycho Educational Profile-Revised (PEP-R; Schopler et al., 1990), or the Leiter International Performance Scale–Revised (Leiter-R; Kaplan & Sacuzzo, 2010). Verbal and performance development were estimated separately, but for each child, an overall developmental quotient was calculated for the time of the assessment. In cases of incomplete assessments (*n* = 27), a best estimate was made from the retrieved information or from structural observations recorded in medical records.

### Postintervention Evaluation

All 94 participants were cognitively assessed or evaluated before starting school at 7 years of age. Cognitive level was assessed with WPPSI-III or the Wechsler Intelligence Scale for Children–Fourth Edition (WISC-IV; Wechsler, 2007), and the school form and level was recorded. A new assessment with ADOS was performed within 1 year after starting school to evaluate the severity of autism symptoms. Both cognitive evaluation and ADOS assessments were performed by ADOS-certified psychologists, blind to the child’s study group belonging (intervention or comparison group).

### Procedures and Analyses

#### Outcome Measurement

All children were assessed with ADOS at baseline as well as at the time of evaluation. The main outcome in the present study was each child’s development. Two different methods were used to assess each child’s development: (1) difference between ADOS raw scores at evaluation minus ADOS raw scores at baseline and (2) the corresponding difference for ADOS-calibrated severity score (Gotham et al., 2009). To estimate the change per time unit, the two above mentioned differences were divided by the number of years elapsed between the dates of the baseline assessment and the evaluation.

#### Statistical Methods

The pre- and postintervention demographic characteristics among children in the intervention group were compared with those of the children in the comparison group using χ^2^ tests (dichotomous variables), or Mann-Whitney U tests (continuous variables). Two outcome measurements were considered in the main analyses: Yearly change (=slope) of ADOS total score and yearly change of ADOS severity scores. All slopes were calculated using (scores at evaluation − scores at baseline)/number of years [with three decimals] elapsed between the date of baseline and the date of evaluation. Factors influencing the outcome measurements were first evaluated using univariate analyses of variance (ANOVAs; binary factors) or linear regression analyses (continuous factors). All factors with *p* values below 0.2 were included in the final analysis of covariance (ANCOVA) models to evaluate the effect of the intervention (NDBI) independent from putative confounders. In addition to the main analyses, mixed effect models were carried out to estimate the individual, and the study group mean, respectively, slope of the change of ADOS total scores between the baseline and evaluation date. Any positive change (yes vs. no) of ADOS module between baseline and evaluation among children in the intervention group was compared with that of children in the comparison group using multivariable linear logistic regression analysis. Statistics were performed using IBM SPSS statistics Version 23, and Gauss (GaussTM, Aptech Systems Inc., Maple Valley, WA, USA; http://www.aptech.com). *P* values less than .05 were regarded to be statistically significant.

#### Ethics

The study was approved by the Regional Ethics Committee at Lund University, Lund, Sweden. All parents of children included in the study have given their informed consent to the study, and all data were confidential and anonymous.

## Results

### Study Group Characteristics

The study group characteristics are displayed in Table 1. More than 80% of the children in both study groups were males, more than 90% of them were born in Sweden, and most of them had mothers who were born in any of the Nordic countries. Children in the intervention group were enrolled at an earlier age (*p* < .001) and more often suffered from developmental delay (*p* = .003) than children in the comparison group. The age at evaluation and other baseline characteristics were similar between the study groups.

**Table 1.** Baseline Characteristics of Children in the Intervention and Comparison Groups.

	Intervention group, *n* = 67		Comparison group, *n* = 27		*p* value for difference between groups
	*n*/*Mdn*	%/[IQR]	*n*/*Mdn*	%/[IQR]	
Baseline characteristics					
Year of birth					
2003	2	3			.125^a^
2004	12	17.9	3	11.1	
2005	24	35.8	13	48.1	
2006	15	22.4	10	37.0	
2007	14	20.9	1	3.7	
Male gender	55	82.1	22	81.5	.945^a^
Not born in Sweden	5	7.5	2	7.4	.993^a^
First child	34	50.7	12	44.4	.580^a^
Non-Nordic mother	24	35.8	7	25.9	.356^a^
Enrollment					
Age at enrolment, *Mdn* [quartiles]	4.0	[3.2-4.7]	4.9	[4.1-5.7]	<.001^b^
Developmental scores, *Mdn* [quartiles]	65	[47-77]	80	[68-100]	.001^b^
Developmental delay (IQ < 70)	40	59.7	7	25.9	.003^a^
IQ < 55	23	34.3	4	14.8	.018^a^
IQ 55-69	17	25.4	3	11.1	
IQ 70-84	15	22.3	8	29.6	
IQ ≥ 85	12	17.9	12	44.4	
Age at first ADOS (baseline), median [quartiles]	3.9	[3.1-4.6]	4.8	[4.0-5.6]	
Intervention					
Age at first intervention, median [quartiles]	4.7	[4.0-5.4]			
Duration of intervention, median [quartiles]	1.9	[1.4-2.4]			
Evaluation					
Age at evaluation, median [quartiles]	8.0	[7.6-8.4]	8.1	[7.7-8.6]	.362^b^
Developmental scores, median [quartiles]	76	[55-90]	85	[60-100]	.171^b^
Developmental delay (IQ < 70)	27	40.3	9	33.3	.530^a^
School level					
Common school	2	3.0	3	11.1	.565^a^
Common school, assistance needed	31	46.3	12	44.4	
Common school, special group	11	16.4	5	18.5	
Special school, high level	8	11.9	2	7.4	
Special school, low level	15	22.4	5	18.5	

*Note*. IQR = interquartile range; IQ = intelligence quotient; ADOS = Autism Diagnostic Observation Schedule.

a *p* value obtained from χ^2^ test. ^b^*p* value obtained from Mann–Whitney *U* test.

#### Individual and Weighted Mean ADOS Total Score Change Between Baseline and Evaluation

**Figure 2.**
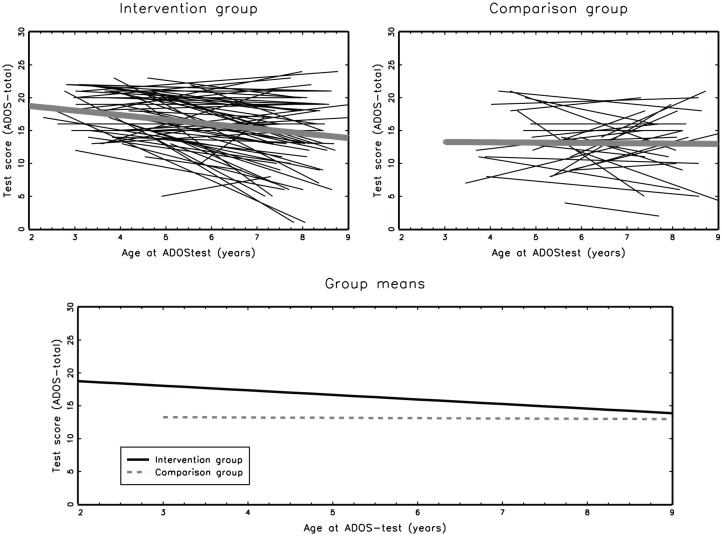
Autism Diagnostic Observation Schedule (ADOS) total test scores by age and study group. The groups mean slopes between baseline and evaluation tests were obtained using mixed effect models.

Figure 2 shows the ADOS total scores by study group and age at ADOS test. For each study group, the weighted mean slope was obtained using mixed effects models, taking all individual changes into consideration. A significant improvement of the ADOS scores was detected in the intervention group (*p* < .001), whereas no such improvement was seen in the comparison group (*p* = .872). Among children with developmental delay, mixed effect models revealed a significant improvement of ADOS-R scores between baseline and evaluation (slope: −0.5, 95% CI [−0.9, 0.0], *p* = .030) among children in the intervention group, whereas no such improvement was detected among children with developmental delay in the comparison group (slope 0.0, 95% CI [−0.9, 0.9], *p* = .979).

#### Change of ADOS Total and Severity Scores by Study Group: The Main Results From Multivariable Analyses

Table 2 shows the baseline ADOS-R scores (total and severity scores, respectively) at baseline, evaluation, and the score change (per 12 months) by study group and child characteristics. The impact of the investigated factors on score change was first evaluated using univariate analyses (ANOVA for class variables and linear regression analysis for the continuous variable “age at ADOS baseline”). The univariate analyses revealed that children in the intervention group reduced their ADOS-R total scores significantly compared with children in the comparison group and that gender, maternal country of birth, or age at ADOS baseline were not associated with ADOS-R total score change. A weak association was indicated between developmental delay and increased ADOS-R total scores, and absence of older siblings and reduced ADOS-R total scores, respectively. These two latter factors (both factors with *p* < .2 in the univariate analyses) were regarded as possible confounders in the investigation of a possible effect of the intervention on ADOS total score changes and were therefore included in the final ANCOVA multivariable model. Adjustment for these two mentioned factors slightly strengthened the association between intervention and an improvement (reduction) of ADOS total scores between baseline and evaluation. For ADOS severity scores, the univariate analysis indicated (*p* = .06) a larger improvement in children in the intervention group compared with those in the comparison group, but this indicated association disappeared when maternal parity and age at baseline assessment were adjusted for in the final ANCOVA (both factors with *p* < .2 in the univariate analyses). The ADOS severity score changes were of the same magnitude among children with developmental delay as among typically developed children (*p* = .762).

**Table 2.** ADOS Scores at Baseline, at Evaluation, and Score Change (per 12 Months), for ADOS Total Score and Calibrated Severity Scores, Respectively.

	Baseline ADOS score		Evaluation ADOS score		Time between baseline and evaluation	Score change (per 12 months)		Change difference between groups, univariate models		Change difference between groups, multivariable model	
	*M*	*SD*	*M*	*SD*	*M* (years)	*M*	95% CI	Difference	*p*	Difference	95% CI
ADOS total scores											
Intervention group	17.5	3.7	14.4	5.7	4.0	**−0.8**	**[−1.2, −0.4]**	**−0.9**	**.019**	**−1.1**	**[−1.9, −0.4]**
Comparison group	13.0	4.5	13.1	5.3	3.3	0.1	[−0.7, 0.9]	Reference		Reference	
Possible confounders											
Males	16.3	4.6	14.0	5.8	3.8	−0.6	[−1.0, −0.2]	−0.1	.805	—	
Females	15.8	3.7	14.4	4.7	3.9	−0.5	[−1.0, 0.0] *ns*	Reference			
Only, or first child	16.9	3.5	13.8	5.6	3.8	−0.9	[−1.3, −0.4]	−0.6	.103	−1.1	[−1.2, 0.1]
Older siblings	15.6	5.1	14.4	5.6	3.8	−0.3	[−0.8, 0.3]	Reference		Reference	
Developmental delay	17.6	4.1	15.9	5.4	3.9	−0.3	[−0.8, 0.1]	0.5	.195	**0.8**	**[0.1, 1.5]**
No developmental delay	14.9	4.4	12.2	5.1	3.5	**−0.8**	**[−1.3, −0.2]**	Reference		Reference	
Non-Nordic mother	17.2	4.2	14.4	5.6	4.1	**−0.8**	**[−1.2, −0.3]**	−0.3	.424		
Mother born in a Nordic country	15.8	4.5	13.9	5.6	3.7	−0.5	[−0.9, 0.0] *ns*	Reference			
Age at ADOS baseline (per 1-year increment)	—	—	—	—		—	—	0.1	.533		
ADOS severity scores											
Intervention group	7.7	1.6	6.6	2.2	4.0	**−0.2**	**[−0.4, −0.1]**	−0.3	.050	−0.3	[−0.5, 0.2]
Comparison group	6.1	1.9	6.3	2.4	3.3	0.1	[−0.2, 0.4]	Reference		Reference	
Possible confounders											
Males	7.2	1.9	6.4	2.3	3.8	−0.2	[−0.4, −0.0]	−0.1	.549	—	
Females	7.4	1.6	7.2	1.9	3.9	−0.1	[−0.2, 0.1]	Reference			
Only, or first child	7.6	1.3	6.4	2.2	3.8	−0.3	[−0.5, −0.1]	−0.3	.066	−0.3	[−0.6, 0.2]
Older siblings	7.0	2.2	6.7	2.3	3.8	0.0	[−0.3, 0.2]	Reference			
Developmental delay	7.6	1.8	6.6	2.1	3.9	−0.2	[−0.4, 0.0]	0.0	.762	—	
No developmental delay	7.0	1.8	6.5	2.4	3.5	−0.1	[−0.4, 0.1]	Reference			
Non-Nordic mother	7.3	1.8	6.2	2.3	4.1	−0.3	**[−0.5, 0.0]**	0.2	.310		
Mother born in a Nordic country	7.3	1.8	6.7	2.2	3.7	−0.1	[−0.3, 0.1]	Reference			
Age at ADOS baseline (per 1-year increment)	—	—	—	—		—	—	**0.2**	**.024**	0.1	[−0.0, 0.3]

*Note*. ADOS = Autism Diagnostic Observation Schedule; *ns* = not significant; ANCOVA = analysis of covariance. Results obtained from *t* tests (univariate analyses), linear regression (association between age at base line and score change), and ANCOVA (adjusted results). The adjusted models included factors with *p* values < .2 in the univariate analyses. Significant associations (*p* < .05) are in boldface.

#### Change of ADOS Module

Table 3 shows the ADOS modules by time point (baseline or evaluation, respectively) and study group. Children with positive module changes represents children who were tested with a more advanced ADOS module at evaluation than at baseline. In the intervention group, 39 children (58%), compared with 14 children (54%) in the comparison group, were tested with a higher module at evaluation than at baseline. No significant association between module change and study group was identified (Table 3).

**Table 3.** ADOS Module Used at Baseline and at Evaluation, Respectively, and ADOS Module Change Between Assessments.

Study group	Baseline ADOS module (rows)	Total	Evaluation ADOS module (columns)			Module changes^a^				Odds ratio for any change of module (intervention vs. comparison group)	
						Positive change		No change			
			1	2	3	*n*	%	*n*	%	Crude OR	Adjusted^b^ OR [95% CI]
Intervention group	1	44	18	13	13	39	58.2	28	41.8	1.2, *p* = .7	2.0 [0.7, 6.0], *p* = .2
	2	23	0	10	13						
	3	0	0	0	0						
Comparison group	1	9	5	2	2	14	53.8	12	46.2		
	2	17	0	7	10						
	3	1	0	0	1						

*Note.* ADOS = Autism Diagnostic Observation Schedule. Odds ratios (OR) were obtained using univariate and multivariable logistic regression analyses.

a Changes of ADOS module used at baseline and at evaluation. ^b^Adjusted for developmental delay and ADOS module at baseline (first child, and age at ADOS baseline, *p* = .3 and *p* = .6, respectively, were initially included, but finally excluded from the multiple model).

## Discussion

The current study found that children in the intervention group improved their ADOS-R total scores between baseline and evaluation. Such an improvement (lower ADOS total scores at evaluation than at baseline) was not detected among children in the comparison group. For ADOS-R total, the change was significantly more apparent among children in the intervention group than among children in the comparison group. For ADOS severity scores, no such difference between the study groups was detected. Children in the intervention group improved their ADOS-R total score even in the presence of developmental delay but to a somewhat lower degree than children without any intellectual disability. The change of ADOS severity scores between base line and evaluation was not influenced by developmental level.

### Comparison With Previous Studies

NDBI-designed treatment models have frequently demonstrated significant social emotional development and language gains in toddlers and infants with ASD. The Early Start Denver Model (ESDM) was specifically developed to meet the needs of infants and toddlers with ASD in “naturalistic” settings (Rogers & Dawson, 2010). In a randomized controlled study (Dawson et al., 2010), 48 children with ASD were followed under a 2-year intervention program. The ESDM group showed a significantly higher increase in cognitive, language, social, and adaptive behavior compared with children in a community-based program (Dawson et al., 2010; Dawson et al., 2012). Yet another randomized trial, the JASPER study (Shire et al., 2017), showed significant larger treatment effect for the JASPER group in measure of the children’s joint engagement, social communication, and play. No differences between the groups could be found in the children’s development of receptive or expressive language. The rather short intervention period (10 weeks) and evaluation at exit and 1 month later restrict the possibility to draw conclusions of maintaining effects from this study. Not all intervention studies have found significant effects of ABA-based intervention programs. In a naturalistic Swedish study (Fernell et al., 2011), 208 children with ASD in Stockholm County were followed over a 2-year period. The children were engaged in high-intensive, low-intensive, or targeted ABA-based intervention. All children improved when evaluated with the Vineland Adaptive Behavior Scale (Sporrow et al., 2005), especially among children with normal cognitive functioning, but no difference was found between the intensive and nonintensive group (Fernell et al., 2011).

### Strengths and Limitations

The current observational study was performed using the child’s home and preschool support. The baseline diagnostic validity was ensured since all children in the current study were diagnosed with autistic disorder (F 84.0) according to ADOS-R and Autism Diagnostic Interview (Lord et al., 1994)—criteria estimated by multidisciplinary teams. The cognitive levels at baseline were estimated for each child using a variety of instruments (see Material and Method).

In the current study, ADOS-R was the only instrument available to measure autism symptoms and general development. In a systematic review, evaluating tools to measure outcome for young children with ASDs, McConachie et al. (2015) found no single instrument to be adequately validated to ensure a fair description of the children’s developmental outcome after interventions. It was concluded that although the Vineland Adaptive Behavior Scale is frequently used as a tool for global measure of function, there is little evidence of its use in young children with ASD. The same review discussed the ADOS as an instrument to describe and measure restricted and repetitive behavior as well as social/communicative development, and the review reported that the ADOS has been used to evaluate developmental outcome in 14 observational studies and 11 intervention evaluation studies (McConachie et al., 2015). In many of these recent studies, the ADOS was one of the instruments used to measure improvement of autism symptoms after miscellaneous intervention programs. Thus, in the absence of suitable instruments to measure general development among children with autism, the ADOS test may be an adequate choice even though the ADOS change is not optimal for evaluating improvement of communicative and social skills. The basic idea behind the ADOS scoring system is the axiom that the severity of autism in a certain child is constant even if the communicative and verbal skills improve with age. Experiences from previous clinical studies have shown that ADOS scores, in the original form, are not that constant (Shumway et al., 2012). The fact that ADOS total scores have shown to decrease with increasing social and verbal skills makes them likely to be influenced by the NDBI program, if the program truly improves social interaction.

To achieve consistency of ADOS scores when verbal skills improve, the ADOS severity scores have been developed (Gotham et al., 2009). Thus, even if the NDBI program really would have a true impact on improvement of communicative skills, the treatment would not result in decreasing ADOS severity scores at evaluation. These assumptions are in concordance with the results from the present study, in which an association between NDBI and improvement of ADOS-R total scores was detected, whereas no such association was indicated for ADOS severity scores.

The study had the advantage that all children with baseline data were included—and were not selected as often is the case in randomized trials. One weakness is that the intervention and comparison groups differed regarding age at enrollment, developmental quotient at baseline, and severity of ASD symptoms. These factors were adjusted for in the analyses, but it could not be ruled out that residual confounding remains due to undetected selection bias. It would not be possible to perform a randomized trial in the Skåne area as the current intervention program has been offered to all children with ASD and their families since 2004. As the NDBI program was offered to all children with ASD and their parents, the comparison group consisted of children of parents who did not accept the offer. As most parents accepted the offer, the comparison group was considerably smaller than the intervention group, a fact that markedly decreased the power to detect a true treatment effect. The reasons for parents not to accept the offered interventions varied, but it seems reasonable to assume that those parents differed from the intervention group regarding their resources to give their children the needed support and encouragement at home. These factors may influence child development and might be potent confounders in the current study. It is likely that parents who accepted to participate in the NDBI program would have been more prone to activate their children at home than parents who declined the offering, regardless of the NDBI program. Thus, the results from the current study do not necessarily only evaluate the current specified NDBI program but could also be a proxy for the beneficial effect of activating children with autism per se. The presence of such an effect could be regarded as an interesting finding in itself. In spite of the difficulties to evaluate an existing NDBI program, it is of uttermost importance to evaluate the interventions as they appear in community and clinical settings—not as they would have appeared during optimal conditions. The lack of information regarding the type of services received by children in the comparison might influence the degree of generalizability of the findings in the current study. This question could not be fully addressed as the size of the effect difference between groups is strongly dependent on the magnitude of the difference between the services received by the intervention group and the services received by the comparison group. However, it seems probable that the habilitation services received by the current comparison group are similar to interventions offered in other national, regional, or local settings. Thus, it seems likely that the finding of a positive effect of the NDBI program in Skåne could be generalized to other areas with similar community services for children with ASD. An advantage compared with many randomized trials is that the current study evaluated the long-term beneficial effects of the intervention program, which is important given the extreme and rising costs for intense treatment programs.

### Implications and Recommendations

The results from the current study must be cautiously interpreted, but they do support the previous studies that have reported on significant improvements of autism symptoms after participation in intensive comprehensive intervention programs. The results also suggest that children with developmental delay could benefit to a similar degree as other children. The current study has obvious disadvantages but also advantages as it has shown effects of an intervention program in a true clinical setting. This fact might be of immense importance when health authorities are about to distribute public funds for children with special needs.
